# Fundamental and frontier research of immune responses to influenza vaccines in human aging: from cross-sectional and longitudinal studies to clinical trials and the geroscience perspective

**DOI:** 10.1186/s12979-023-00392-2

**Published:** 2023-11-29

**Authors:** Sean X. Leng, Albert C. Shaw

**Affiliations:** 1 Division of Geriatric Medicine and Gerontology, Department of Medicine, Johns Hopkins Center on Aging and Immune Remodeling, Department of Molecular Microbiology and Immunology, Johns Hopkins University School of Medicine, Johns Hopkins Bloomberg School of Public Health, JHAAC Room 1A.38A, 5501 Hopkins Bayview Circle, Baltimore, MD 21224 USA; 2 Section of Infectious Diseases, Department of Internal Medicine, Yale University School of Medicine, New Haven, Connecticut, USA

Seasonal influenza is a common respiratory viral infection with the highest burden of severe disease and death affecting older adults worldwide. Annual immunization is the cornerstone for influenza prevention. However, despite the availability of influenza vaccines specially formulated for older adults, vaccination coverage varies widely among different countries/regions and older adults continue to suffer disproportionally high morbidity and mortality from seasonal influenza. Therefore, further understanding of and improving influenza immunization for older adults remain a priority in translational aging research. The seven original research articles and one review in this Topical Collection cover a number of fundamental and frontier research topics relevant to influenza immunization research in human aging. The reported research employs a variety of approaches, from cross-sectional and longitudinal cohort analyses, vaccine immunogenicity studies, to clinical trials and the geroscience perspective.

Studies by Wunderlich et al. [1] and Shapiro et al. [2] are based on data from the Johns Hopkins Longitudinal Influenza Immunization Study of Aging in adults 75 years of age and older (JH LIISA 75+), an ongoing longitudinal cohort since 2014 specifically designed to study influenza immunization in community-dwelling older adults [3]. The study by Wunderlich, et al. [1] aimed at addressing pre-existing humoral immunity as measured by influenza strain-specific hemagglutination-inhibition (HAI) antibody titers at the beginning of each seasonal epidemic and interseason waning of humoral immunity, both of which are critically important for better understanding of vaccine-induced immune protection against influenza in the highly vaccinated, but still vulnerable older adult population. The authors took advantage of available data on pre-vaccination HAI antibody titers measured using both current and prior season vaccine strain antigens among JH LIISA 75 + study participants enrolled for multiple consecutive influenza seasons in which virus strains were changed in the vaccine formula. The results showed that interseason waning of HAI antibody titers was evident with significant variations among vaccine strains and study seasons. In addition, the conventional approach, i.e., measuring pre-vaccination HAI antibody titers using current season vaccine strain antigens, appeared to underestimate residual HAI antibody titers from prior season vaccination and, thus, overestimate interseason waning. Moreover, interseason waning and prior season post-vaccination HAI antibody titers had significant and independent impact on pre-existing humoral immunity. Shapiro, et al. [2] analyzed the data collected over four influenza seasons (2019–2022) of JH LIISA 75 + for potential intersection of biological sex and gender in contributing adverse events (AE) following vaccination. The results provide preliminary evidence supporting a larger role for biological sex than for gender in contributing to AE and suggested hormonal mechanisms that may mediate this sex difference.

In a small cross-sectional observation study, Frasca and colleagues [4] report that obesity, similar to aging, is associated with increased circulating inflammatory cytokines, but reduced antibody response to influenza vaccine as well as defects in B cells as indicated by higher expression levels of senescence markers and T-bet and lower levels of E47. An exciting aspect of this study is that a 12-month voluntary weight reduction program significantly attenuated such effects in obese individuals, begging for large-scale studies to confirm these findings and further investigations into mechanistic pathways.

Xiao et al. [5] report that a quadrivalent inactivated influenza vaccine was immunogenic and well-tolerated among young and older adults living in Tianjin, China. However, older adults had significantly poorer HAI antibody response to IIV4 than young individuals, implicating age-related immunosenescence as a risk factor. This is in the context of a country with the largest and fastest growing older adult population and yet extremely low influenza vaccination coverage [6]. Studies like this may serve as a rationale and evidence to support and promote influenza vaccination coverage for older adults in China and other countries/regions with low or no influenza vaccination coverage [6]. It should be noted that vaccination, not vaccine itself, saves lives.

The three clinical trials reported by Liu et al. [7], Schmader and colleagues [8], and Martin et al. [9] are equally fascinating. The IMPROVE trial conducted by Liu et al. evaluated the potential impact of circadian rhythm on vaccine-induced antibody responses in middle-aged and older adults. The results indicate better influenza vaccine immunogenicity in older adults and women when the vaccine was administered in the morning [7]. Morning vaccination is a simple and cost-free intervention that is feasible in most healthcare systems. Data from the trial comparing immunogenicity of trivalent adjuvanted inactivated influenza vaccine (aIIV3) vs. high dose vaccine (HD IIV3) in older adults reported by Schmader and colleagues [8] demonstrate overall similar HAI antibody responses. For the primary outcome, the aIIV3 seroconversion rate for H3N2 did not meet noninferiority criteria compared with HD-IIV3, but the HD-IIV3 seroconversion rate was not statistically significantly superior to aIIV3 either. Both HD-IIV3/4 and aIIV3/4 are preferentially recommended for older adults. Martin et al. [9] report data from a pilot geroscience-guided trial of metformin treatment in nondiabetic older adults demonstrating a significant increase in HAI antibody response to HD-IIV3 with post-vaccination trending increases in circulating Tfh and reduced expression of the exhaustion marker CD57 in CD4+ T cells. Finally, the review article by Cadar et al. [10] provides, from a geroscience perspective, an in-depth discussion on targeting the hallmarks of aging, such as inflammageing, cellular senescence, immunometabolism dysregulation, microbiome disturbance, etc., to improve immune responses to influenza vaccines in older adults.

Overall, the collection of these original articles and review offers new data and perspective, albert preliminary in some cases, addressing several critically important and yet basic and cutting-edge aspects of immune responses to influenza vaccines in older adults, such as vaccine immunogenicity, B and T cell responses, waning of vaccine-induced humoral immunity, and impact of host factors (e.g., sex, circadian rhythm, obesity) as well as potential interventions with weight reduction or senolytics. By shedding light on these fundamental and frontier topics, this thematic series aims to spark innovation, foster collaboration, and stimulate further research that will lead to the development of more effective immunization strategies for the protection of the vulnerable older adult population against influenza.
